# Inference of Gene Regulatory Networks from Genetic Perturbations with Linear Regression Model

**DOI:** 10.1371/journal.pone.0083263

**Published:** 2013-12-23

**Authors:** Zijian Dong, Tiecheng Song, Chuang Yuan

**Affiliations:** 1 School of Electronic Engineering, Huaihai Institute of Technology, Lianyungang, Jiangsu, China; 2 School of Information Science and Engineering, Southeast University, Nanjing, Jiangsu, China; 3 Department of Health Technology and Informatics, The Hong Kong Polytechnic University, Hang Kong, China; Leibniz-Institute for Farm Animal Biology (FBN), Germany

## Abstract

It is an effective strategy to use both genetic perturbation data and gene expression data to infer regulatory networks that aims to improve the detection accuracy of the regulatory relationships among genes. Based on both types of data, the genetic regulatory networks can be accurately modeled by Structural Equation Modeling (SEM). In this paper, a linear regression (LR) model is formulated based on the SEM, and a novel iterative scheme using Bayesian inference is proposed to estimate the parameters of the LR model (LRBI). Comparative evaluations of LRBI with other two algorithms, the Adaptive Lasso (AL-Based) and the Sparsity-aware Maximum Likelihood (SML), are also presented. Simulations show that LRBI has significantly better performance than AL-Based, and overperforms SML in terms of power of detection. Applying the LRBI algorithm to experimental data, we inferred the interactions in a network of 35 yeast genes. An open-source program of the LRBI algorithm is freely available upon request.

## Introduction

Exploring the structure of Gene Regulatory Networks (GRN) is a key element in understanding gene functions, especially in some complex diseases [1]–[3]. Direct experimental methods to explore the relationships among genes are time-consuming and labor-intensive. Statistical inference on GRN is a process of identifying gene interactions from limited experimental data using computational analysis, and is much more efficient.

Several models have been applied to describe the GRN. An intuitive and frequently applied method is to model the GRN as graphs [4]–[6], where the genes are considered as nodes and the interactions among them represented as edges. Several graphical methods, including directed acyclic graphs and directed cyclic graphs, have been proposed in [7]–[9]. GRN can also be modeled by the graphical Gaussian model [10], or the Bayesian network model [11]. Information theory, for instance, mutual information and synergy, can be also used to infer the GRN [12], [13]. Due to high measurement cost of gene chip technology, only limited number of samples can be obtained. This limitation may result in low inference accuracy when applying synergy or mutual information to analyze the GRN.

In the last decade, Structural Equation Modeling (SEM) [14] has been used to infer GRN [9], [15], [16]. Exploiting genetic perturbation data and gene expression data, the work in [16] used SEM model via an adaptive Lasso based algorithm (AL-Based) to infer the networks. With simulations, the authors showed that the AL-based method had better performance than all other existing methods. With the two same types of data, Cai et. al. introduced a sparse SEM model, and stated that their Sparsity-aware Maximum Likelihood (SML) algorithm significantly outperformed all other algorithms, including the AL-based one [17], [18].

In this paper, we also study the gene regulatory networks with SEM model using both genetic perturbation data and gene expression data, and transfer the SEM to a Linear Regression (LR) model through matrix transformation. In this transformation process, regulatory information in GRN will not be lost. Instead of ML approaches or classic Lasso methods, we propose an approach to infer the networks via the LR model by using a Bayesian method (LRBI). Simulations show that our LRBI algorithm is effective and reliable, and offers significantly better performance than the AL-based algorithm. Compared with SML, LRBI has significantly better performance in terms of power of detection, but has slightly worse performance in false discovery rate. LRBI also has the advantages that the estimation of the initial parameters and the consideration of the data sensitivity are not needed.

## Model and Methods

### The LR model for gene network inference

We consider 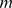 genes, 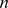 individuals' measurement using microarray. Without loss of generality, we assume that there are 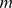 makers. As in [9], [16], [17], the GRN obeys the form of SEM, where genes are the nodes, and interactions among genes are the edges, i.e.
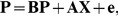
(1)where 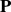 is an 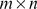 matrix, 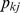 is the 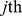 expression level of the 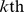 gene; 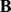 is an 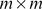 matrix, defining the structure of the gene regulatory networks, 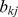 is the regulatory effect of the 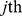 gene on the 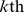 gene; 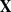 is an 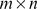 matrix, 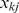 is the genotype of the 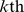 marker in the 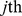 perturbation; 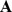 is an 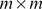 matrix representing the effect of each eQTL; 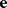 is an 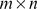 matrix, and 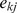 is the 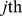 measurement noise of the 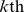 gene. All elements in 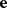 are independent and identically distributed (i.i.d).

We assume that there is no self-loop of each gene, so that all diagonal entries of 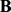 are zeros. We also assume that each gene has its own corresponding QTL, and the loci of the 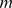 eQTLs have been determined by an existed method, but the effects of these eQTLs are unknown yet. Therefore 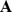 has 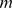 unknown entries, and all other entries are zeros. Without loss of generality, we assume that all the unknowns in 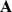 are the diagonal entries.

With the predetermined eQTLs matrix 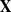 and the gene expression data 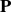, the inference for GRN is to determine the unknown entries of 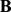 and 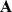 with appropriate optimization methods.

Since all the unknown parameters are in 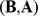, (1) can be written as follows
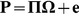
(2)where 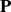 is still the 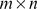 matrix defined above, 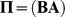, 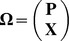. We further rewrite (2) to 
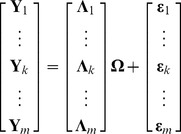
(3)where 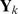 is the 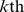
row of 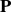, 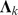 is the 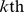
row of 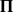, 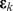 is the 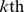
row of 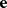.

By the definition and the structure of (3), we can infer the parameters row by row. Therefore, the problem can be decomposed into
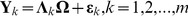
(4)


In (4), the parameters that need to be inferred are 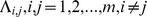, and 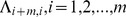.

### Bayesian inference for the LR models

In gene regulatory networks, most entries of 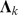 are zeros, so 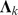 is sparse. Therefore, we assume that all entries of 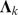 follow Gaussian distribution with mean zeros. We also assume that entries of 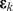 are i.i.d, and normally distributed with mean zeros and variance 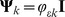, where 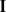 is an 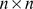 identity matrix.

With known 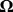, the parameters to be estimated in (4) are 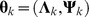. The joint prior distribution can be factorized as:
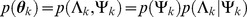
(5)


Rewriting (5) leads to
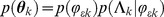
(6)


We assume that 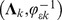 has a joint prior distribution of Gaussian-Gamma [19], with 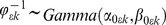
(7)

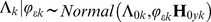
(8)


where 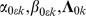 are hyper parameters, should be preset to fixed values. 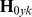 is a symmetric positive definite matrix. We will set it to an identity matrix in the implementation of algorithm for simplification.

The likelihood is
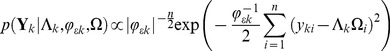
(9)


where 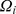 is the 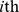 column of 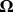. The joint posterior distribution of 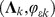 is proportional to the product of the prior and the likelihood
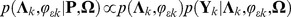
(10)


According to the prior distribution in (7∼8) and the likelihood in (9), the joint posterior distribution (10) can be written as
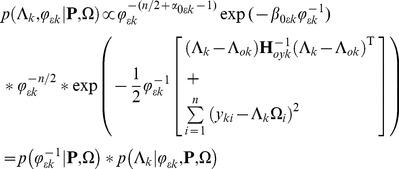
(11)


where
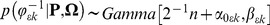
(12)

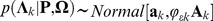
(13)


and
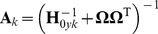
(14)

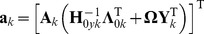
(15)

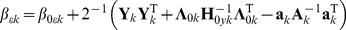
(16)


More details of the derivation can refer to Text S1. A similar result was shown in [14], [20], where (12∼16) were parts of an iterative process to solve the Confirmatory Factor Analysis (CFA) model.

We can sample the posterior distributions in (12) and (13) to constitute an iterative process. Since the values in (14)∼(16) are all determined, the parameters of the Gamma distribution in (12), 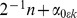 and 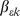, are all fixed. As a result, the posterior distribution of 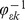 will not be affected by the samples of 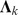. It is noted that only 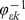,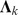 can be sampled, therefore, the iterative process may be not effective and accurate. Thus, we modify the calculations of 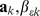 in (15) and (16), and substitute 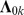 by 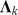.
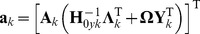
(17)

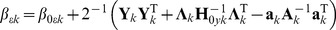
(18)


The combination of (12, 13) and (17, 18) forms an iterative process. We execute this iterative process with a sufficient number of times, and until a steady state is reached. A sequence of sets of 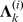 are obtained by sampling from the posterior distribution in (13), which are then averaged to get the estimated parameters of 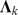. To get accurate results, we must guarantee that the iteration reaches its steady state. A simple stopping condition is to test the value of the square difference of the inferred parameters between two successive iterations, i.e. 
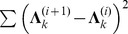
. If the difference is small enough (say 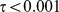), the iteration has reached a stable state. The choice of 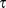 can influence the accuracy. The smaller 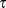 is, the higher the accuracy of the parameter approximation is, naturally at the cost of more iterations and increased computational time.

The sketch of an algorithm is as follows:

*Input the eQTLs matrix 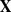, and the gene expression data 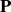; Set the initial hyperparameters 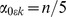, 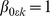, 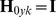. 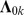 is set to a 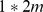
vector, where only the 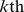 entry is 1, all other entries are zeros. 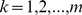; Assign a small value to 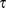.*

*For 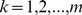
*

*Calculate 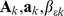
by (14∼16);*

*Repeat:*

1. *Get the sample of 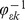 from the Gamma distribution by (12);*
2. *Get the sample of 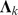 from the Normal distribution by (13);*
3. *Calculate 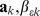 by (17)(18);*
4. *Calculate 
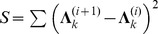
;*
5. *If 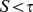,then end the iteration, else go to step 1;*

*End for*

More details of the algorithm implementation can refer to the software package F1.

It should be noted that it would be better to choose the second half of the samples and average them to get accurate result. The reason is that at the beginning of the iteration, the gap between the estimated 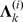 and the true values is large.

## Results

### Simulations

Let 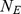 be the number of edges in 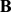 (the original network), 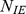 be the number of edges in 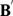 (the inferred network), 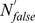 be the number of edges which exist in 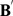 but not in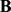, 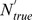 be the number of edges which exist in both 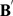 and 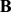, therefore 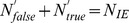. Define Power of Detection 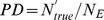, and False Discovery Rate 
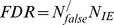
.

Logsdon and Mezey [16] had shown that the AL-based algorithm outperformed the PC-algorithm [21], [22], the QTLnet algorithm [23], and had comparable performance with the QDG algorithm [24]. Cai et al. stated that their SML algorithm offered significantly better performance than the AL-based algorithm and the QTL algorithm in PD and FDR [17], [18]. Therefore, we shall compare our LRBI algorithm with SML and AL-based.

Firstly, we carried out simulations following the setups in [16]. We simulated two types of directed acyclic gene networks: one with 10 genes and the other one with 30 genes. Averaged 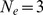 edges were created per gene, which meant that there were on average 3 edges created between one gene and all other genes. If an edge existed from node 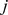 to node 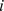, then 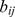 was sampled from a uniform distribution on the interval 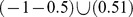; otherwise 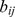 was set to 0. Entries of 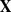 took values from the set 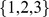
with the corresponding probabilities 0.25, 0.5, and 0.25 respectively. Each gene has its own corresponding QTL, and 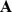 is assumed to be an identity matrix. Each entry of 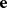 in (1) was sampled from a Gaussian distribution 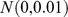. 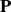 was calculated by (1).

We generated cyclic or acyclic networks for simulations, and used LRBI to infer the parameters of the simulated networks. For cyclic networks,LRBI can obtain the steady-state solutions naturally. By inference, the steady regulatory relations can be got, if some cyclic regulatory relations among genes existed.

Due to the inference characteristic of Bayesian methods, the estimated parameters are not regressed to zeros as in Lasso methods. Therefore, an edge from gene 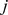 to gene 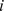 is considered to be present if 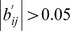, otherwise, there is no edge from gene 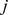 to gene 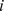.

**Figure 1:**
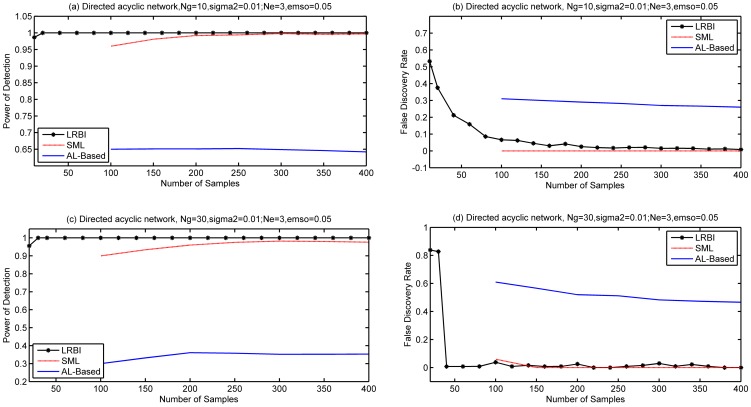
Performance of LRBI for directed acyclic networks. The performance of SML and AL-Based algorithms is also shown for comparisons. The average number of edges per node is 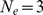, the variance of noise is 0.01, and no edge exists if 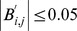 for decision. (a) and (b) are for a gene network of 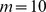, (c) and (d) are for a gene network of 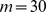.

Simulation results for the setups described above are shown in Figure 1, where (a) and (b) are for the gene network of 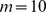, (c) and (d) are for the gene network of 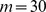. LRBI has a better performance than SML in terms of PD, but SML outperforms LRBI algorithm in terms of FDR. Both LRBI and SML significantly outperform the AL-Based algorithm in terms of PD and FDR. The PD of LRBI reaches 1 when the number of samples is 20 or more for both the two scenarios 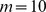 and 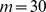.

**Figure 2:**
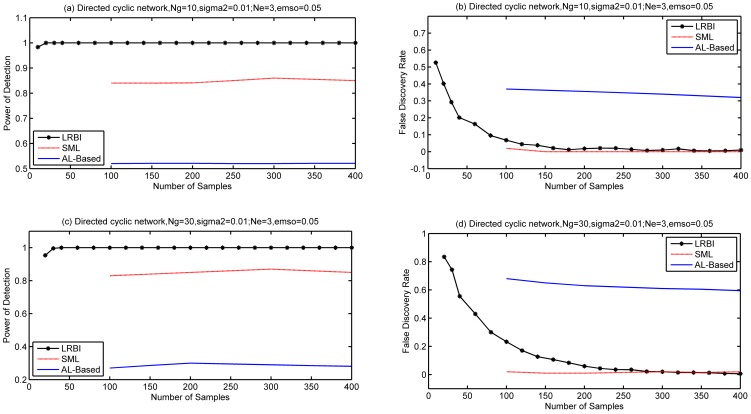
Performance of our LRBI algorithms for directed cyclic networks. The performance of SML and AL-Based algorithms is also shown for comparisons. The average number of edges per node is 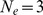, the variance of noise is 0.01, and no edge exists if 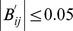 for decision. (a) and (b) are for a gene network of 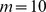, (c) and (d) are for a gene network of 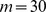.

Secondly, we simulated two types of directed cyclic gene networks: one with 10 genes and the other one with 30 genes. Averaged 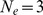 edges were created per gene. We employed the same procedure used in the acyclic scenario to generate 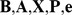. Simulation results are shown in Figure 2, where (a) and (b) are for the gene network of 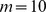, (c) and (d) are for the gene network of 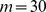. LRBI has significantly better performance than SML in terms of PD, and SML outperforms LRBI algorithm in terms of FDR. When the number of samples is large enough, the FDRs of LRBI and SML are all close to zeros. Both LRBI and SML significantly outperform the AL-Based algorithm in PD and FDR.

**Figure 3:**
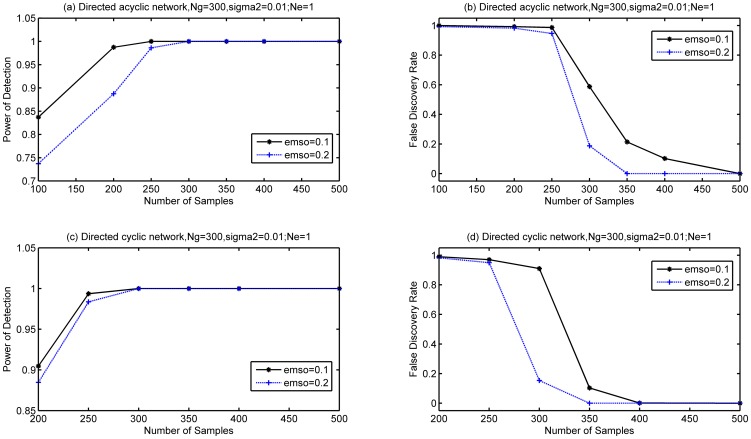
Performance of LRBI algorithms for various decision thresholds. Two network cases are simulated to find the impact of decision thresholds on PD and FDR. (a) and (b) are for directed acyclic networks, (c) and(d) are for directed cyclic networks; 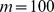,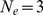, the variance of noise is 0.01. Thresholds are 0.05, 0.1, 0.2 (emso in figure).

Thirdly, we simulated the impact of different decision thresholds on performances. We used a bigger network 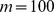. Averaged 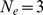 edges were created per gen, and the variance of noise was 0.01. Three decision thresholds 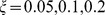 were simulated. In simulations, if we found that 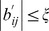, then we set 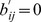. A directed acyclic network and a directed cyclic network were simulated and the results were separately shown in Figure 3 (a) (b) and Figure 3 (c) (d).

**Figure 4:**
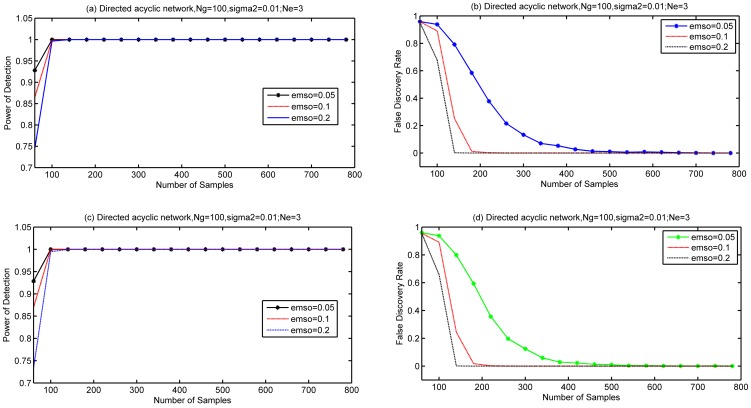
Performance of LRBI algorithms for various decision threshold with 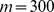. Two network cases are simulated to find the impact of decision thresholds on PD and FDR. (a) and (b) are for directed acyclic networks, (c) and(d) are for directed cyclic networks; 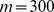, 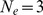, the variance of noise is 0.01.Thresholds are 0.1 and 0.2.

We continued the simulations with a even bigger network with 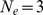, 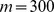 to study the impact of decision thresholds on performance. The variance of noise was 0.01. Two decision thresholds 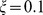 and 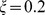 were simulated respectively. Both directed acyclic network and directed cyclic network were simulated, and the results were separately shown in Figure 4 (a) (b) and Figure 4 (c) (d). As confirmed by Figure 3 and Figure 4, a large decision threshold can reduce the FDR, but it also lowers the PD. Therefore, the decision thresholds used in simulations or applications should be chosen carefully.

**Figure 5:**
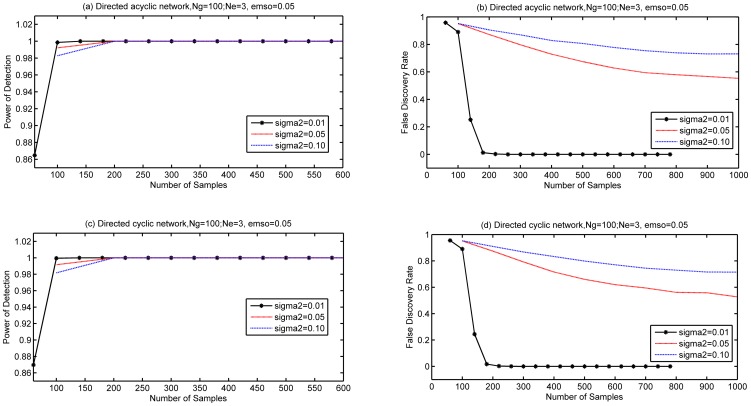
Performance of LRBI algorithm under various noise levels. Two network cases are simulated to find the impact of noise level on PD and FDR. (a) and (b) are for directed acyclic networks, (c) and (d) are for directed cyclic networks; 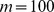, Decision thresholds is 0.05. Three noise levels are simulated.

Finally, we evaluated the impact of noise levels on the performance of LRBI. Here, we used networks with 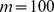, 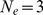. Again, we applied LRBI to both directed acyclic and directed cyclic networks. The variance of noise was set to 0.01, 0.05, and 0.1 respectively. The simulation results are shown in Figure 5, where (a) and (b) are for directed acyclic network, (c) and (d) are for directed cyclic network. We find that the PD performance is always excellent, but the FDR of LRBI is worse when the noise level increases, even when the number of samples is relatively large.

We have stated that LRBI cannot infer parameters to zeros automatically, but can infer them with high precision. In most of the simulations we conducted, the decision thresholds are 0.05. That is to say, if the value inferred is lower than 0.05, the entry is considered to be zero. This implies that the numerical difference between the inferred value and the original value is less than 0.05 for most of the entries in regulatory networks. We define that numerical difference as 
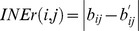
. Through simulations, we found that 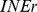 was also very small for the entry whose value is nonzero in the original network. This feature is very meaningful, because the inferred parameters can accurately indicate the regulatory relationships among genes. An acyclic network was simulated, with 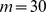,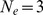, sigma2 = 0.01, decision threshold 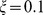. Some results are shown in Table 1.

**Table 1:** Some INErs of a network inferred by LRBI.

(i,j)	(1,8)	(1,17)	(1,26)	(1,28)	(2,17)	(2,19)	(3,1)	(3,5)	(3,23)	(3,28)	(4,2)	(4,12)
B(i,j)	0.9749	0.8016	−0.5050	−0.9123	−0.9925	0.9274	−0.7733	0.8633	0.5397	0.9918	0.8308	−0.6049
B'(i,j)	0.9301	0.7725	−0.4812	−0.8379	−0.9913	0.8959	−0.7452	0.8412	0.5183	0.9577	0.8081	−0.5974
INEr(i,j)	0.0448	0.0291	0.0238	0.0744	0.0012	0.0315	0.0281	0.0221	0.0214	0.0341	0.0227	0.0075

The network is an acyclic network with m = 30, Ne = 3, sigma2 = 0.01, decision threshold is 0.1.

### Case study

Here, we applied LRBI to infer the gene regulatory networks using the gene expression data and the genetic makers, which were assayed in 112 segregants of a cross between the yeast strains BY4716 and RM11-1a [25]. The cross had 5727 genes with small number of samples, so a pretreatment process was needed to select strong cis-eQTLs and interactions among genes [16]. We dealt with the filtered dataset provided by Logsdon [16], in which only 35 genes were used. The 35 yeast genes are SEO1, NUP60, RCY1, IRC18, TPK3, PHD1, JLP1, SNF7, PCD1, RPL19A, SEN1, OST6, BUB2, BUL1, PHA2, ORC5, FYV6, SLM2, HAL9, RDL1, POC4, ASA1, ECM13, TYR1, RNQ1, SFA1, PRM7, SAN1, HIM1, YEL073C, SAP1, SNZ3, MST27, YHR054C, DAL7.

**Figure 6:**
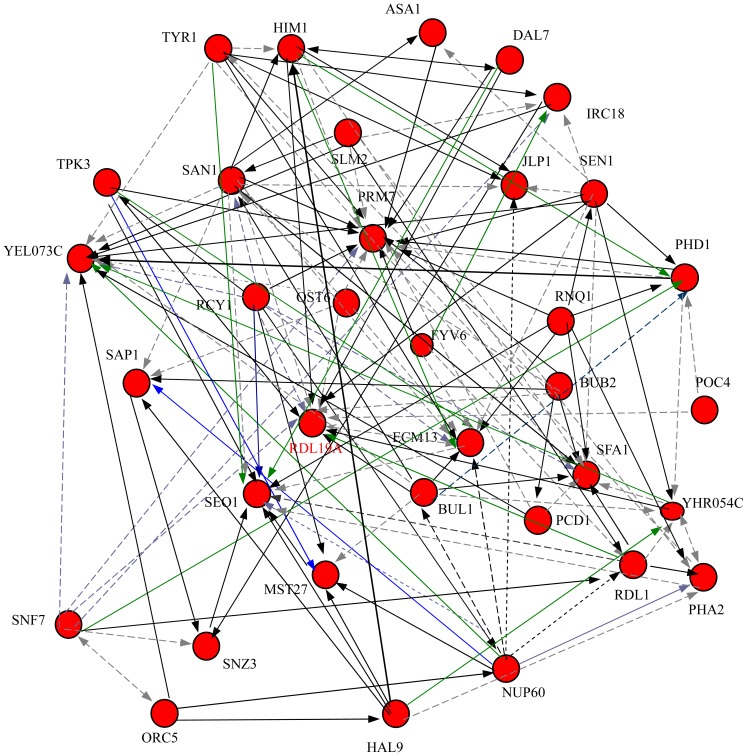
Regulatory network reconstruction for the 35 genes. These genes are filtered out by the methods as in [16]. In the figure, a solid line denotes that the interaction between two genes is positive regulatory, while a dotted line denotes a negative regulatory. Some color lines are used to make the figure clear. There are 145 regulator–target pairs, among which, 78 pairs are positive regulations, and 67 pairs are negative regulations.

With 112 samples for these 35 genes, and the eQTLs data, we inferred the regulatory network as shown in Figure 6. It is noted that our algorithm doesn't need to assume the network is cyclic or acyclic. There are 145 edges in the inferred network. A total of 31 genes are regulators of at least one target, and 32 genes have at least one regulator. A total of 28 genes occur both as regulators and targets.

There were only 4 instances of reciprocal regulation (two genes act on each other) presented: 
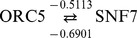
, 
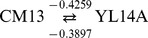
, 
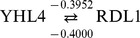
, 
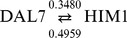
.

Among the 145 regulator–target pairs, there are 78 positive regulations, and 67 negative regulations. To verify the inference result, we used the Generate Regulation Matrix tool in the website of YEASTRACT [26] to create the gene regulatory network with the 35 selected yeast genes described above. In the network generated by the tool, there are only three regulatory relationship, HIM1 regulated by HAL9 [27], YEL073C regulated by PHD1 [27], and FYV6 regulated by PHD1 [28]. From the experimental yeast data, we deduced two out of the three regulatory relationships, HIM1 regulated by HAL9, YEL073C regulated by PHD1.

## Conclusion and Discussion

We modeled the gene regulatory networks by using a LR model, and proposed a Bayesian method to complete the inference. We conducted a series of simulations to evaluate the performance of the proposed algorithm LRBI, and compared LRBI with another two algorithms, the AL-Based and the SML algorithms. LRBI had a significantly better performance than AL-based regarding to both PD and FDR. Compared to SML, LRBI showed a better performance in PD and slightly worse in FDR. This feature of LRBI makes more sense. Considering two cases, one is that we can find less false edges but loss more true edges, the other one is that we can find more, or even all true edges among genes, but with slightly more false edges, the latter one is more meaningful.

The proposed algorithm was accurate, and the gap between the inferred and the original parameters was less than 5% (even 2%) in most case. The proposed algorithm was also very effective. We inferred the GRN of the 35 yeast genes in a short time (1.2 seconds in a laptop), while for the SML algorithm, a program error occurred after about 52 minutes' run with the same 35 yeast genes data set. LRBI also had the benefit that the dependency of the performance on the estimates of initial parameters is not strong. For simplicity, we just assign some constants to these parameters in simulations and case studies. Therefore, the LR model and the LRBI algorithm can provide an effective way of exploiting both gene expression and perturbation data to infer GRN.

The reason our method seemed to perform better was that, LRBI fully exploited the structure of the SEM, and transformed it into a linear regression model without information loss, while AL-Based only partly exploited the structure of the SEM and used the adaptive Lasso to infer the networks, so LRBI was more effective. LRBI used the Bayesian method, while SML essentially used the maximum likelihood method to infer the GRN, therefore SML was not efficient and sensitive to data. However, there are many other methods for linear regression problems, such as hierarchical Bayesian, variational approximation, and so on. These methods can potentially improve the inference accuracy of GRN with the linear regression model proposed by this paper.

However, the FDR of LRBI is considerably high when the noise level is large, and another issue is the ability of dealing with large-scale gene networks. Thus, a future work is to decrease FDR in high-noise context, and apply new strategies to handle large-size gene networks.

## Supporting Information

Text S1 **Bayesian Inference for the Linear Regression Model.**
(PDF)

Software S1 **Software package implementing the LRBI algorithm.**
(RAR)
