# Supplementary material for: Inference of Gene Regulatory Networks from Genetic Perturbations with Linear Regression Model
Source: PLoS One. 2013 Dec 23;8(12):e83263. doi: 10.1371/journal.pone.0083263 (PMC3871530; doi:10.1371/journal.pone.0083263)
Supplement: Text S1 — Bayesian Inference for the Linear Regression Model. (PDF) [file pone.0083263.s001.pdf]

Inference of Gene Regulatory Networks from Genetic Perturbations with Linear Regression Model
Supporting text S1

Zijian Dong [1, 2]*, Tiecheng Song [2] , Chuang Yuan [3]

1. School of Electronic Engineering, Huaihai Institute of Technology, Lianyungang 222005, China;
2. School of Information Science and Engineering, Southeast University, Nanjing 210096, China;
3. Department of Health Technology and Informatics, The Hong Kong Polytechnic University, Hang Kong, China.

Bayesian Inference for the Linear Regression Model

We use the model defined in (4)

$$\mathbf{Y}_k = \mathbf{\Lambda}_k \mathbf{\Omega} + \boldsymbol{\varepsilon}_k, \quad k = 1, 2, ..., m \tag{A.1}$$

$\mathbf{\Lambda}_k$ is sparse in GRN, therefore, $\mathbf{\Lambda}_k$ follow Gaussian distribution with mean zero. $\boldsymbol{\varepsilon}_k$ is i.i.d, and normally distributed with mean zeros and variance $\mathbf{\Psi}_k = \varphi_{\varepsilon k}\mathbf{I}$, where $\mathbf{I}$ is an $n \times n$ identity matrix.

With known $\mathbf{\Omega}$, the parameters to be estimated in (A.1) are $\boldsymbol{\theta}_k = (\mathbf{\Lambda}_k, \mathbf{\Psi}_k)$. The joint distribution can be factorized as:

$$p(\boldsymbol{\theta}_k) = p(\mathbf{\Lambda}_k, \mathbf{\Psi}_k) = p(\mathbf{\Psi}_k)\, p(\mathbf{\Lambda}_k \mid \mathbf{\Psi}_k) \tag{A.2}$$

Rewriting (A.2) leads to

$$p(\boldsymbol{\theta}_k) = p(\varphi_{\varepsilon k})\, p(\mathbf{\Lambda}_k \mid \varphi_{\varepsilon k}) \tag{A.3}$$

We assume that $(\mathbf{\Lambda}_k, \varphi_{\varepsilon k}^{-1})$ has a joint prior distribution of Gaussian-Gamma, with

$$\begin{aligned} \varphi_{\varepsilon k}^{-1} &\sim Gamma(\alpha_{0\varepsilon k}, \beta_{0\varepsilon k}) \\ &= \varphi_{\varepsilon k}^{-(\alpha_{0\varepsilon k}-1)} \exp(-\beta_{0\varepsilon k}\varphi_{\varepsilon k}^{-1}) \end{aligned} \tag{A.4}$$

$$\begin{aligned} \mathbf{\Lambda}_k \mid \varphi_{\varepsilon k} &\sim Normal(\mathbf{\Lambda}_{0k}, \varphi_{\varepsilon k}\mathbf{H}_{0yk}) \\ &= \varphi_{\varepsilon k}^{-n/2} \exp\left[-\frac{1}{2}\varphi_{\varepsilon k}^{-1}(\mathbf{\Lambda}_k - \mathbf{\Lambda}_{0k})\mathbf{H}_{oyk}^{-1}(\mathbf{\Lambda}_k - \mathbf{\Lambda}_{0k})^{\mathbf{T}}\right] \end{aligned} \tag{A.5}$$

where $\alpha_{0\varepsilon k}, \beta_{0\varepsilon k}, \mathbf{\Lambda}_{0k}$ are hyper parameters. $\mathbf{H}_{0yk}$ is a symmetric positive definite matrix.

The likelihood is

$$p\left(\mathbf{Y}_k \mid \mathbf{\Lambda}_k, \varphi_{\varepsilon k}, \mathbf{\Omega}\right) \propto \left|\varphi_{\varepsilon k}\right|^{-\frac{n}{2}} \exp\left(-\frac{\varphi_{\varepsilon k}^{-1}}{2}\sum_{i=1}^{n}\left(y_{ki}-\mathbf{\Lambda}_k\Omega_i\right)^2\right) \tag{A.6}$$

where Ω_i is the ith column of $\mathbf{\Omega}$. The joint posterior distribution of $\left(\mathbf{\Lambda}_k, \varphi_{\varepsilon k}\right)$ is proportional to the product of the prior and the likelihood

$$p\left(\mathbf{\Lambda}_k, \varphi_{\varepsilon k} \mid \mathbf{P}, \mathbf{\Omega}\right) \propto p\left(\mathbf{\Lambda}_k, \varphi_{\varepsilon k}\right) p\left(\mathbf{Y}_k \mid \mathbf{\Lambda}_k, \varphi_{\varepsilon k}, \mathbf{\Omega}\right) \tag{A.7}$$

According to the prior distribution and the likelihood, the joint posterior distribution can be written as

$$\begin{aligned} &p\left(\mathbf{\Lambda}_k, \varphi_{\varepsilon k} \mid \mathbf{P}, \mathbf{\Omega}\right) \propto \varphi_{\varepsilon k}^{-(n/2+\alpha_{0\varepsilon k}-1)} \exp(-\beta_{0\varepsilon k}\varphi_{\varepsilon k}^{-1}) \\ &*\varphi_{\varepsilon k}^{-n/2} * \exp\left(-\frac{1}{2}\varphi_{\varepsilon k}^{-1}\left[\left(\mathbf{\Lambda}_k - \mathbf{\Lambda}_{ok}\right)\mathbf{H}_{oyk}^{-1}\left(\mathbf{\Lambda}_k - \mathbf{\Lambda}_{ok}\right)^{\mathrm{T}} + \sum_{i=1}^{n}\left(y_{ki}-\mathbf{\Lambda}_k\Omega_i\right)^2\right]\right) \end{aligned} \tag{A.8}$$

We calculate the bracketed term in (A.8),

$$\begin{aligned} I &= \left(\mathbf{\Lambda}_k - \mathbf{\Lambda}_{ok}\right)\mathbf{H}_{oyk}^{-1}\left(\mathbf{\Lambda}_k - \mathbf{\Lambda}_{ok}\right)^{\mathrm{T}} + \sum_{i=1}^{n}\left(y_{ki}-\mathbf{\Lambda}_k\Omega_i\right)^2 \\ &= \left(\mathbf{\Lambda}_k - \mathbf{\Lambda}_{ok}\right)\mathbf{H}_{oyk}^{-1}\left(\mathbf{\Lambda}_k - \mathbf{\Lambda}_{ok}\right)^{\mathrm{T}} + \sum_{i=1}^{n}\left[y_{ki}^2 + \left(\mathbf{\Lambda}_k\Omega_i\right)^2 - 2y_{ki}\mathbf{\Lambda}_k\Omega_i\right] \\ &= \left(\mathbf{\Lambda}_k - \mathbf{\Lambda}_{ok}\right)\mathbf{H}_{oyk}^{-1}\left(\mathbf{\Lambda}_k - \mathbf{\Lambda}_{ok}\right)^{\mathrm{T}} + \mathbf{Y}_k\mathbf{Y}_k^{\mathrm{T}} + \mathbf{\Lambda}_k\mathbf{\Omega}\mathbf{\Omega}^{\mathrm{T}}\mathbf{\Lambda}_k^{\mathrm{T}} - \mathbf{\Lambda}_k\mathbf{\Omega}\mathbf{Y}_k^{\mathrm{T}} - \mathbf{Y}_k\mathbf{\Omega}^{\mathrm{T}}\mathbf{\Lambda}_k^{T} \\ &= \mathbf{\Lambda}_k\mathbf{H}_{oyk}^{-1}\mathbf{\Lambda}_k^{\mathrm{T}} + \mathbf{\Lambda}_k\mathbf{\Omega}\mathbf{\Omega}^{\mathrm{T}}\mathbf{\Lambda}_k^{\mathrm{T}} + \mathbf{Y}_k\mathbf{Y}_k^{\mathrm{T}} + \mathbf{\Lambda}_{0k}\mathbf{H}_{oyk}^{-1}\mathbf{\Lambda}_{0k}^{\mathrm{T}} \\ &\quad -\mathbf{\Lambda}_k\mathbf{H}_{oyk}^{-1}\mathbf{\Lambda}_{0k}^{\mathrm{T}} - \mathbf{\Lambda}_k\mathbf{\Omega}\mathbf{Y}_k^{\mathrm{T}} - \mathbf{\Lambda}_{ok}\mathbf{H}_{oyk}^{-1}\mathbf{\Lambda}_k^{\mathrm{T}} - \mathbf{Y}_k\mathbf{\Omega}^{\mathrm{T}}\mathbf{\Lambda}_k^{T} \end{aligned} \tag{A.9}$$

Let $\mathbf{A}_k = \left(\mathbf{H}_{0yk}^{-1} + \mathbf{\Omega}\mathbf{\Omega}^{\mathrm{T}}\right)^{-1}$, then

$$\begin{aligned} I &= \mathbf{\Lambda}_k\mathbf{A}_k^{-1}\mathbf{\Lambda}_k^{\mathrm{T}} + \mathbf{Y}_k\mathbf{Y}_k^{\mathrm{T}} + \mathbf{\Lambda}_{0k}\mathbf{H}_{oyk}^{-1}\mathbf{\Lambda}_{0k}^{\mathrm{T}} \\ &\quad -\mathbf{\Lambda}_k\mathbf{A}_k^{-1}\left[\mathbf{A}_k\left(\mathbf{H}_{oyk}^{-1}\mathbf{\Lambda}_{0k}^{\mathrm{T}} + \mathbf{\Omega}\mathbf{Y}_k^{\mathrm{T}}\right)\right]^{\mathrm{T}} - \left[\left(\mathbf{H}_{oyk}^{-1}\mathbf{\Lambda}_k^{\mathrm{T}} + \mathbf{Y}_k\mathbf{\Omega}^{\mathrm{T}}\right)\mathbf{A}_k\right]\mathbf{A}_k^{-1}\mathbf{\Lambda}_k^{\mathrm{T}} \end{aligned} \tag{A.10}$$

Let $\mathbf{a}_k = \left[\mathbf{A}_k\left(\mathbf{H}_{0yk}^{-1}\mathbf{\Lambda}_{0k}^{\mathrm{T}} + \mathbf{\Omega}\mathbf{Y}_k^{\mathrm{T}}\right)\right]^{\mathrm{T}}$, then

$$\begin{aligned} I &= \mathbf{\Lambda}_k\mathbf{A}_k^{-1}\mathbf{\Lambda}_k^{\mathrm{T}} - \mathbf{\Lambda}_k\mathbf{A}_k^{-1}\mathbf{a}_k^{\mathrm{T}} - \mathbf{a}_k\mathbf{A}_k^{-1}\mathbf{\Lambda}_k^{\mathrm{T}} + \mathbf{\Lambda}_{0k}\mathbf{H}_{oyk}^{-1}\mathbf{\Lambda}_{0k}^{\mathrm{T}} + \mathbf{Y}_k\mathbf{Y}_k^{\mathrm{T}} \\ &= \left(\mathbf{\Lambda}_k - \mathbf{a}_k\right)\mathbf{A}_k^{-1}\left(\mathbf{\Lambda}_k - \mathbf{a}_k\right)^{\mathbf{T}} + \mathbf{\Lambda}_{0k}\mathbf{H}_{oyk}^{-1}\mathbf{\Lambda}_{0k}^{\mathrm{T}} + \mathbf{Y}_k\mathbf{Y}_k^{\mathrm{T}} - \mathbf{a}_k\mathbf{A}_k^{-1}\mathbf{a}_k^{\mathrm{T}} \end{aligned} \tag{A.11}$$

In the calculation, we use the symmetric properties of $\mathbf{H}_{oyk}^{-1}$ and $\mathbf{A}_k^{-1} = \mathbf{H}_{0yk}^{-1} + \mathbf{\Omega}\mathbf{\Omega}^{\mathrm{T}}$.

The joint posterior distribution can be written as

$$
\begin{aligned}
&p\left(\mathbf{\Lambda}_k,\varphi_{\varepsilon k}\mid\mathbf{P},\mathbf{\Omega}\right)\propto\varphi_{\varepsilon k}^{-(n/2+\alpha_{0\varepsilon k}-1)}\exp(-\beta_{0\varepsilon k}\varphi_{\varepsilon k}^{-1})\\
&*\varphi_{\varepsilon k}^{-n/2}\exp\left(-\frac{1}{2}\varphi_{\varepsilon k}^{-1}\left[\left(\mathbf{\Lambda}_k-\mathbf{\Lambda}_{ok}\right)\left(\mathbf{H}_{oyk}^{-1}\right)\left(\mathbf{\Lambda}_k-\mathbf{\Lambda}_{ok}\right)^{\mathrm{T}}+\sum_{i=1}^{n}\left(y_{ki}-\mathbf{\Lambda}_k\Omega_i\right)^2\right]\right)\\
&=\varphi_{\varepsilon k}^{-(n/2+\alpha_{0\varepsilon k}-1)}\exp(-\beta_{0\varepsilon k}\varphi_{\varepsilon k}^{-1}-\frac{1}{2}\varphi_{\varepsilon k}^{-1}\left(\mathbf{\Lambda}_{0k}\mathbf{H}_{oyk}^{-1}\mathbf{\Lambda}_{0k}^{\mathrm{T}}+\mathbf{Y}_k\mathbf{Y}_k^{\mathrm{T}}-\mathbf{a}_k\mathbf{A}_k^{-1}\mathbf{a}_k^{\mathrm{T}}\right))\\
&*\varphi_{\varepsilon k}^{-n/2}\exp\left(-\frac{1}{2}\varphi_{\varepsilon k}^{-1}\left(\mathbf{\Lambda}_k-\mathbf{a}_k\right)\frac{1}{\mathbf{A}_k}\left(\mathbf{\Lambda}_k-\mathbf{a}_k\right)^{\mathbf{T}}\right)
\end{aligned}
\tag{A.12}
$$

Obviously, it is the product of a Gamma distribution and a Gaussian distribution, so

$$
p\left(\mathbf{\Lambda}_k,\varphi_{\varepsilon k}\mid\mathbf{P},\mathbf{\Omega}\right)=p\left(\varphi_{\varepsilon k}^{-1}\mid\mathbf{P},\mathbf{\Omega}\right)*p\left(\mathbf{\Lambda}_k\mid\varphi_{\varepsilon k},\mathbf{P},\mathbf{\Omega}\right) \tag{A.13}
$$

where

$$
p\left(\varphi_{\varepsilon k}^{-1}\mid\mathbf{P},\mathbf{\Omega}\right)\sim Gamma\left[2^{-1}n+\alpha_{0\varepsilon k},\beta_{\varepsilon k}\right] \tag{A.14}
$$

$$
p\left(\mathbf{\Lambda}_k\mid\mathbf{P},\mathbf{\Omega}\right)\sim Normal\left[\mathbf{a}_k,\varphi_{\varepsilon k}\mathbf{A}_k\right] \tag{A.15}
$$

We rewrite the parameters again as follows:

$$
\mathbf{A}_k=\left(\mathbf{H}_{0yk}^{-1}+\mathbf{\Omega}\mathbf{\Omega}^{\mathrm{T}}\right)^{-1} \tag{A.16}
$$

$$
\mathbf{a}_k=\left[\mathbf{A}_k\left(\mathbf{H}_{0yk}^{-1}\mathbf{\Lambda}_{0k}^{\mathrm{T}}+\mathbf{\Omega}\mathbf{Y}_k^{\mathrm{T}}\right)\right]^{\mathrm{T}} \tag{A.17}
$$

$$
\beta_{\varepsilon k}=\beta_{0\varepsilon k}+2^{-1}\left(\mathbf{Y}_k\mathbf{Y}_k^{\mathrm{T}}-\mathbf{a}_k\mathbf{A}_k^{-1}\mathbf{a}_k^{\mathrm{T}}+\mathbf{\Lambda}_{0k}\mathbf{H}_{0yk}^{-1}\mathbf{\Lambda}_{0k}^{\mathrm{T}}\right) \tag{A.18}
$$
